# Stromal Annexin A2 expression is predictive of decreased survival in pancreatic cancer

**DOI:** 10.18632/oncotarget.22433

**Published:** 2017-11-15

**Authors:** Adrian G. Murphy, Kelly Foley, Agnieszka A. Rucki, Tao Xia, Elizabeth M. Jaffee, Chiung-Yu Huang, Lei Zheng

**Affiliations:** ^1^ The Sidney Kimmel Comprehensive Cancer Center, Johns Hopkins University School of Medicine, Baltimore, Maryland, USA; ^2^ Department of Oncology, Johns Hopkins University School of Medicine, Baltimore, Maryland, USA; ^3^ Zhejiang University School of Medicine, Hangzhou, Zhejiang, China; ^4^ The Skip Viragh Center for Pancreatic Cancer, Johns Hopkins University School of Medicine, Baltimore, Maryland, USA; ^5^ Division of Biostatistics and Bioinformatics, Sidney Kimmel Comprehensive Cancer Center, Johns Hopkins University School of Medicine, Baltimore, Maryland, USA

**Keywords:** stroma, Annexin A2, tumor microenvironment, pancreatic cancer

## Abstract

Pancreatic ductal adenocarcinoma (PDA) is renowned for high rates of metastasis and poor survival. Its notoriously dense fibrotic stroma contributes to chemoresistance. Stromal signaling in PDA is recognized for its multiple roles in regulating tumor invasion and metastasis. However, no stromal biomarker which can predict survival in PDA exists. Annexin A2 (AnxA2) was formerly identified as a metastasis-associated protein in PDA and tumoral overexpression is associated with poor survival. In this study, we examined AnxA2 expression in the tumor microenvironment in a preclinical model of PDA which suggests its role in tumor colonization.

We injected wild-type (KPC) and AnxA2 knockout (KPCA) pancreatic cells into C57BL/GJ (B6) and AnxA2 knockout (KO) mice using the hemi-spleen model and observed their survival. We performed quantitative immunohistochemistry examining stromal AnxA2 expression in 56 patients who had surgically resected PDA and correlated expression with clinical outcomes.

B6 mice injected with KPC cells demonstrated decreased median survival compared to those injected with KPCA cells (90 days vs. not reached, *p* < 0.0001) whereas there was no survival difference in the AnxA2 KO mice (*p* = 0.63). In patient specimens, we found that high stromal AnxA2 expression (≥80th percentile) was associated with significantly reduced disease-free survival (*p* = 0.002) and overall survival (*p* < 0.001). Using multivariate Cox models, there were no significant associations between other clinical covariates apart from high stromal AnxA2 expression.

This study highlights the role that stromal AnxA2 expression plays as a prognostic marker in PDA and its potential as a predictive biomarker for survival outcomes in PDA.

## INTRODUCTION

Pancreatic ductal adenocarcinoma (PDA) is associated with high rates of metastasis, resistance to cytotoxic chemotherapeutic agents and poor 5-year survival rates [1]. The majority of patients (80%) present with inoperable disease and even those who undergo extensive surgical resection typically recur in 80% of cases and die within 5 years of recurrence [2].

PDA is renowned for its dense fibrous stroma, which contains significant cellular heterogeneity and contributes to chemoresistance [3, 4]. Emerging evidence suggests that stromal signaling regulates growth, invasion and metastasis in PDA [5]. There are increasing efforts focusing on targeting the stromal components of PDA that are known to be aberrantly regulated (e.g., hedgehog inhibition and hyaluronan) [6] which have resulted in the increased efficacy of some chemotherapeutics [7]. Specifically, depleting tumor-associated stromal tissue by inhibiting hedgehog signaling in a genetic mouse model of PDA resulted in a transient increase in intratumoral vascular density and intratumoral concentration of gemcitabine [7]. However, inhibiting hedgehog signaling remains controversial because the depletion of stromal cells through hedgehog dependent and independent mechanisms results in the promotion of PDA growth and metastasis in some mouse models [8, 9]. An additional stromal targeting agent, PEGPH20, which is a recombinant human hyaluronidase enzyme that degrades hyaluronan (HA) in the tumor microenvironment (TME), has also been shown to increase drug perfusion in the TME. HA is an extracellular matrix component that contributes to the formation of the dense and fibrous connective tissue that is present in PDA, which results in increased intratumoral pressure in the TME of PDA [10]. Therefore, targeting the breakdown of HA with PEGPH20 has resulted in increased perfusion of gemcitabine into the TME, which resulted in improved survival outcomes [11].

A phase 2 clinical trial involving GVAX (allogeneic granulocyte-macrophage colony stimulating factor-secreting tumor vaccine) identified Annexin A2 (AnxA2) from the sera of patients with PDA, as a metastasis-associated protein. Patients who developed post-vaccination immune responses had improved recurrence-free survival with neoadjuvant and adjuvant GVAX in conjunction with surgical resection [12, 13]. AnxA2 is a calcium-dependent phospholipid binding protein that plays key roles in cellular structure and function including cell division and movement [14, 15]. It is overexpressed in numerous cancer types and is associated with poor prognostic rates in lung and colorectal cancer [16, 17]. AnxA2 forms heterotetramers on the cell surface that bind to tissue-type plasminogen activator to mediate the conversion of plasminogen to plasmin, resulting in extracellular matrix degradation and proteolytic activation of inactive proteases [18, 19]. Phosphorylation of Tyr^23^ of AnxA2 is responsible for the cell-surface localization of AnxA2 and mediating the invasive and metastatic function of PDA cells [12].

AnxA2 has also been shown to regulate the secretion of a class III semaphorins (Sema3D) from PDA cells [20]. It controls the interaction between Sema3D and its receptor plexin D1 (PlxnD1) on the tumor cell surface, thereby promoting tumor invasion and metastasis. AnxA2 has been reported to have 5–15 fold higher levels of expression in pancreatic tumors compared to normal pancreatic cells [21]. AnxA2 overexpression has been associated with resistance to gemcitabine in PDA, and patients with high levels of AnxA2 expression in their tumors have shorter disease-free survival rates and worse overall survival than those with low expression levels [22]. Given the important role of stroma in PDA, we wished to examine the association between stromal expression of AnxA2 and clinical outcomes.

Understanding the complexity of the stromal compartment may provide further insights into the development of PDA, which could identify potential biomarkers for predicting survival for patients with PDA.

## RESULTS

### Annexin A2 plays a vital role in the colonization of metastatic pancreatic cancer

To evaluate the role of AnxA2 in the TME and stroma, KPCA GFP^+^ cells (*KRAS*^G12D^ *TP53*^R172H^ *PDX-1-CRE*^+/+^ *ANXA2*^−/−^) and KPCA cells with re-expression of AnxA2 (KPCA + AnxA2) were injected into animals using the hemi-spleen model (*n* = 10 animals per group) to generate liver metastases [12, 20].

**Figure 1:**
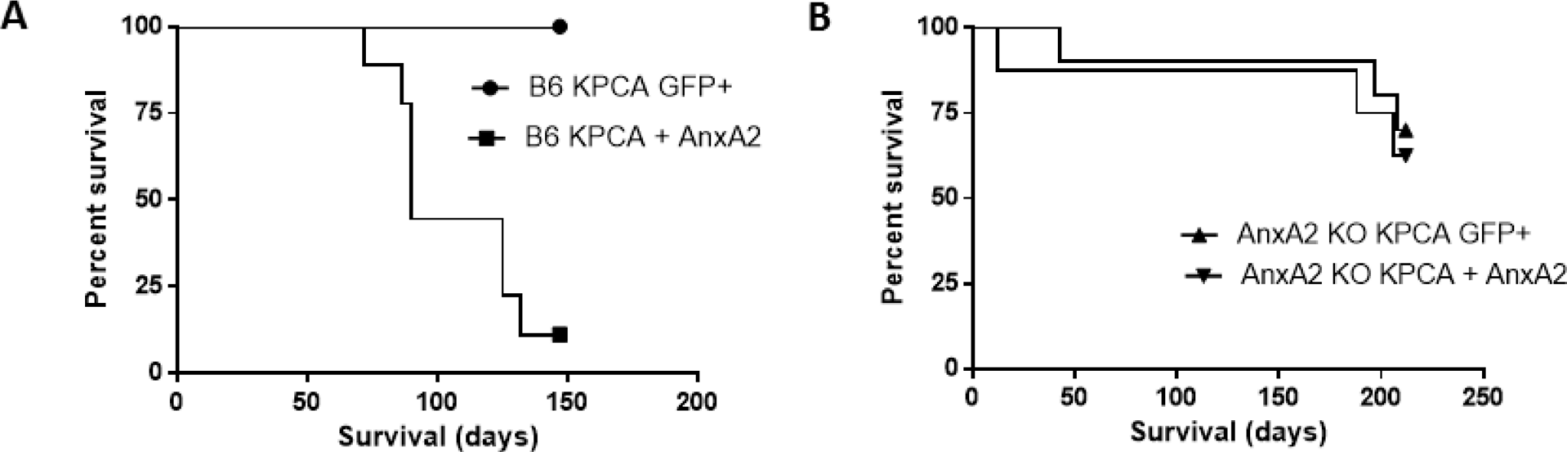
Knock-down of AnxA2 improves survival in the hemi-splenectomy KPC model Kaplan–Meier survival analyses of C57BL/6J (B6) and Annexin A2 knockout (AnxA2 KO) mice injected with KPCA GFP^+^ (AnxA2 deficient) or KPCA +AnxA2 (AnxA2 re-expressed) cells. (**A**) Liver metastases were generated in the hemi-spleen model in B6 where KPCA + AnxA2 cells had shorter survival than KPCA GFP^+^ cells (*p* < 0.001). (**B**) In liver metastases generated in AnxA2 KO mice, there was no significant survival difference between either cell line (*p* = 0.63). 10 mice per group were used in each experiment. Survival curves were compared using log rank test.

Using C57BL/6J (B6) mice, mice receiving KPCA + AnxA2 cells (functionally resembling KPC cells) had decreased survival in the hemi-spleen model compared to mice receiving KPCA GFP^+^ cells (Figure 1A, Supplementary Figure 1, median survival 90 days vs. not reached, *p* < 0.0001). Using the same model in AnxA2 knockout (AnxA2 KO) mice, there was no significant difference in survival when KPCA GFP^+^ or KPCA + AnxA2 cells were used (Figure 1B, Supplementary Figure 1, median survival not reached, *p* = 0.63). These data suggest that AnxA2 expression in the stroma and TME is important for pancreatic tumor homing in the liver (Supplementary Figure 2).

### Clinical characteristics of cohort of patients with resected pancreatic tumors

The clinical characteristics of the patient cohort are summarized in Table 1. The median age of the 56 patients was 62 years (range 43–83). There were more male patients (57.1%) and the majority had positive lymph node status (87.5%). Most tumors had low/intermediate grade differentiation (60%). The majority of the tumors demonstrated perineural invasion (90.7%) and vascular invasion (56.6%), and most patients in our cohort had negative surgical margins (60.7%). The mean stromal AnxA2 score was 0.82 (standard deviation, 0.55; range, 0.02–2.27).

**Table 1:** Clinical characteristics of patients in pancreatic tumor cohort (*N* = 56)

Variable	Value
**Age** – yr.	
Median	62
Range	43–83
**Tumor size** – cm	
Median	3
Range	1–6
**Gender** – no. (%)	
Male	32 (57.1)
Female	24 (42.9)
**Nodal status** – no. (%)	
Positive	49 (87.5)
Negative	7 (12.5)
**Histological grade** – no. (%)	
≥3	22 (40)
≤2	33 (60)
**Perineural invasion** – no. (%)	
Positive	49 (90.7)
Negative	5 (9.3)
**Margin positivity** – no. (%)	
Positive	22 (39.3)
Negative	34 (60.7)
**Vascular invasion** – no. (%)	
Positive	30 (56.6)
Negative	23 (43.4)

A summary of the clinical characteristics of the patients included in our stromal Anx2 database.

### AnxA2 stromal expression is correlated with reduced disease-free survival

The preclinical study showed that stromal AnxA2 expression is important for the homing of the tumors in the liver with subsequent survival benefit. Next we wanted to determine the correlation between AnxA2 expression in the stroma of human PDA and DFS.

**Figure 2:**
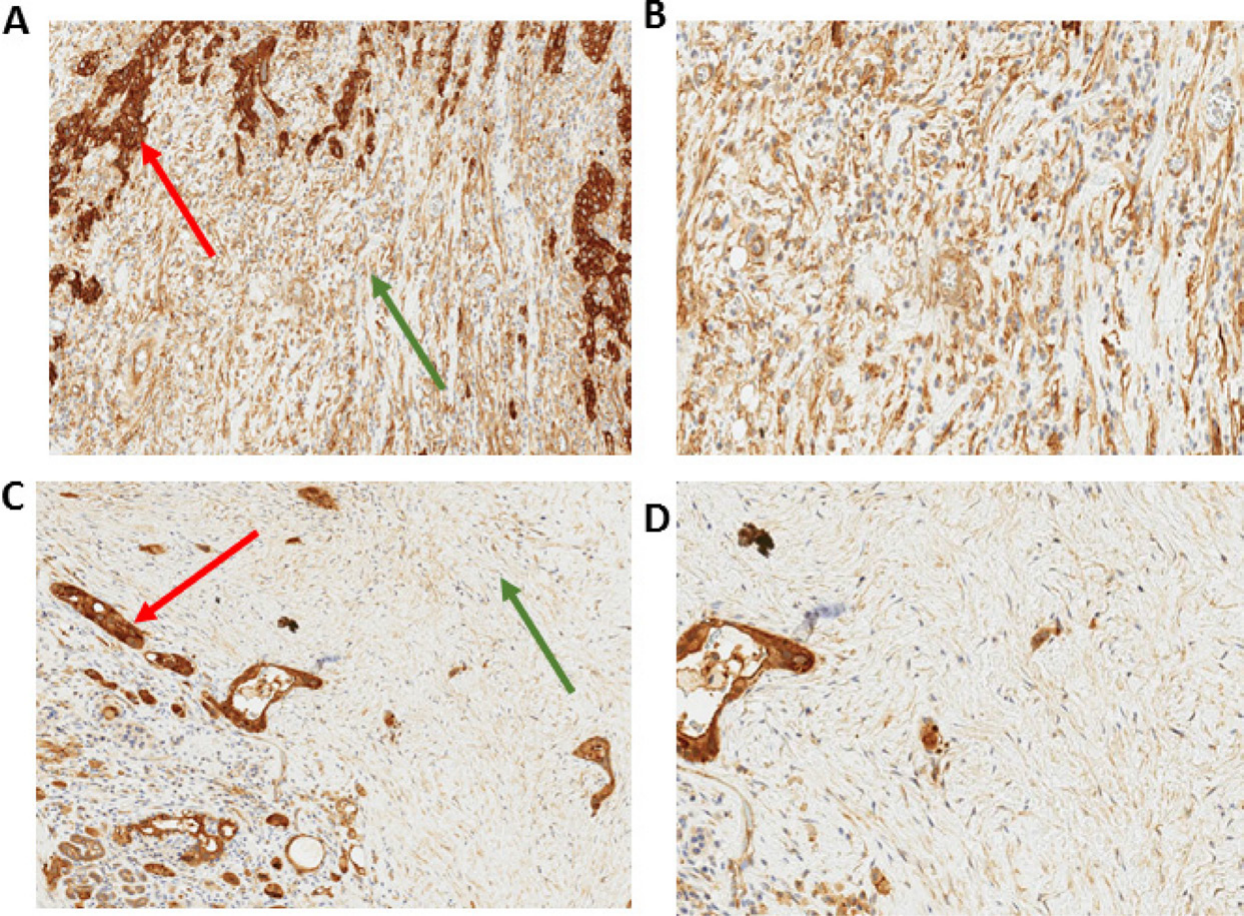
Immunohistochemistry of stromal AnxA2 expression in pancreatic cancer Immunohistochemical staining of AnxA2 expression shows that the subcellular location of AnxA2 in stromal cells is in the cytoplasm of stromal fibroblasts. Representative images of tumor specimen with high stromal AnxA2 expression at 10× magnification (**A**) and 20× magnification (**B**). Representative image of tumor specimen with low stromal AnxA2 expression at 10× magnification (**C**) and 20× magnification (**D**). Red arrows indicate tumor and green arrows indicate stroma.

**Figure 3:**
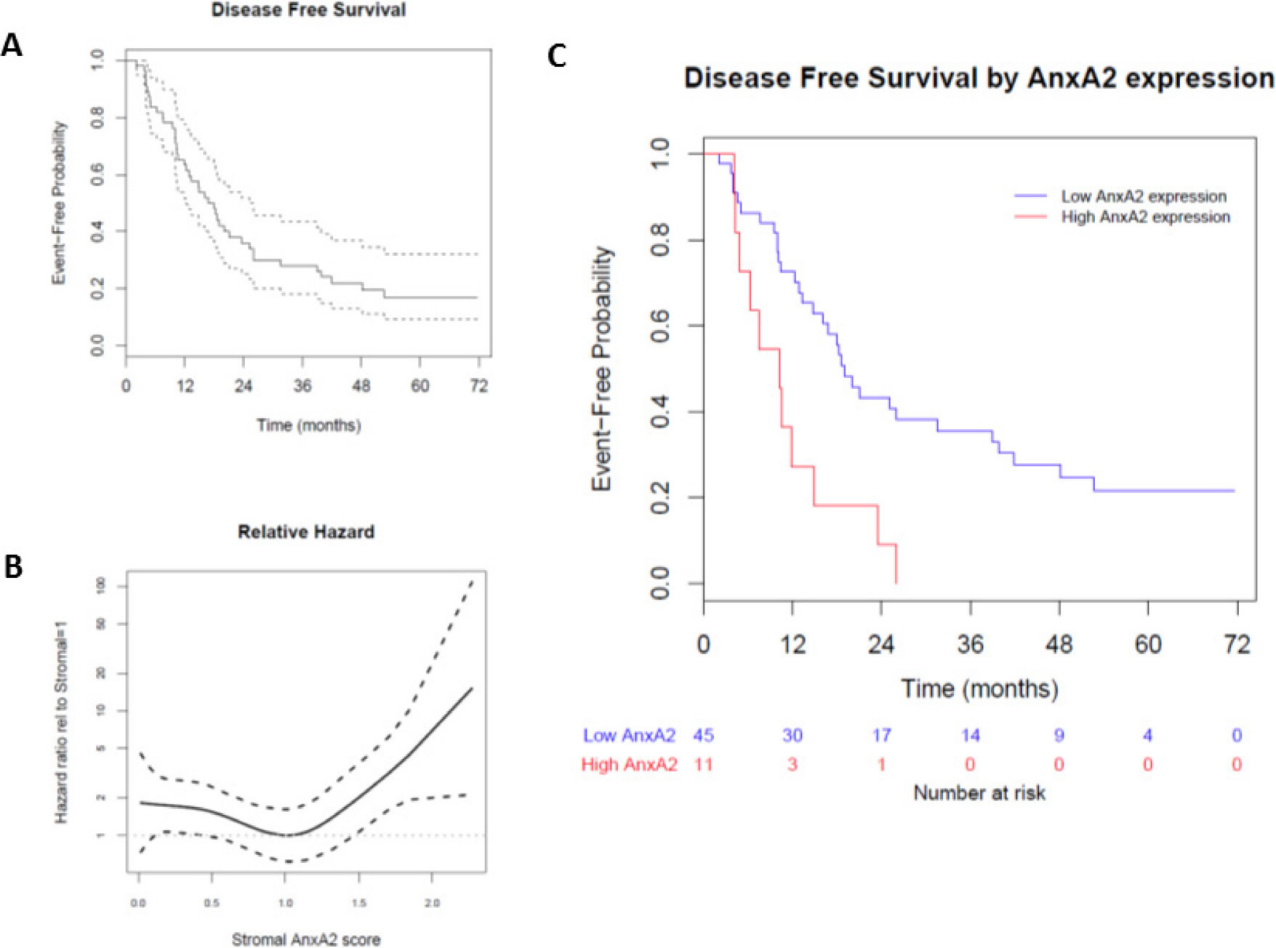
Effect of stromal AnxA2 score on disease-free survival in pancreatic cancer We correlated the disease-free survival rates of the patients in our cohort with stromal AnxA2 expression. (**A**) Kaplan–Meier curve, along with pointwise 95% confidence interval, showing that the median DFS in this cohort is 16.8 months (95% CI 12.3–25.0 months). (**B**) Hazard ratio is estimated by fitting a Cox model with penalized smoothing spline of stromal AnxA2 score as the covariate. The ratio is calculated by dividing the estimated hazard for a given stromal AnxA2 score by that for the stromal AnxA2 score of 1.0. (**C**) Kaplan–Meier curves show that patients with high stromal AnxA2 expression (score > 1.29) have decreased DFS (*p* < 0.001).

We used immunohistochemical staining to quantify stromal AnxA2 expression (Figure 2). Figure 3A shows the Kaplan–Meier curve of DFS for all 56 patients. The estimated median DFS was 16.8 months (95% CI 12.3–25.0 months). Moreover, the progression-free probabilities at 1 and 2 years are 0.63 (95% CI 0.52–0.78) and 0.36 (95% CI 0.25–0.52), respectively. To explore the association between DFS and stromal AnxA2 stromal expression, we fitted a Cox model with penalized smoothing splines, with a degree of freedom of 4, to allow for a nonlinear association between log-hazard of DFS and AnxA2 score. Figure 3B shows the ratio of the estimated hazard function, along with 95% CI, for patients with different values of AnxA2 stromal expression relative to a stromal AnxA2 score of 1. This suggests that the effect was approximately constant for stromal AnxA2 scores smaller than 1 and that the effect increased with AnxA2 scores greater than 1. The lower limit of the 95% CI exceeds 1 for AnxA2 scores greater than 1.5, suggesting that a cutoff value between 1 and 1.5 in AnxA2 score may be used to identify patients at a higher risk of disease progression. When a cutoff value of 1.29 is used (corresponding to the 80th percentile of the observed AnxA2 scores), there was a significant difference in DFS between the high and low AnxA2 expression groups (10.3 vs. 19.0 months, *p* = 0.002) (Figure 3C).

The univariate Cox model estimates a hazard ratio of 2.98 (95% CI 1.45–6.14) comparing patients with and without high AnxA2 stromal expression score (≥1.29). We also used a multivariate Cox model to evaluate the adjusted effect of AnxA2 expression on DFS, adjusting for potential clinical and pathological risk factors, and found that the high AnxA2 stromal expression score (≥1.29) remained significantly associated with DFS (*p* < 0.001) (Table 2).

**Table 2:** Multivariate Cox regression analysis of factors related to disease-free survival

Variable	HR	95% CI	*P* value
AnxA2 score > 1.29	2.52	(1.07, 5.93)	0.03
Age > 60 years	0.94	(0.43, 2.07)	0.87
Female gender	1.94	(0.61, 6.14)	0.26
Tumor size > 3 cm	0.40	(0.11, 1.37)	0.14
Node positive	2.04	(0.64, 6.52)	0.23
Margin positive	0.69	(0.24, 1.99)	0.49
Grade ≥ 3	1.01	(0.47, 2.20)	0.97
Nodes ≥ 3	0.69	(0.27, 1.74)	0.43
Perineural invasion	0.87	(0.23, 3.20)	0.83
Vascular invasion	1.87	(0.77, 4.52)	0.16

Multivariate Cox modelling showing that clinical characteristics, except for high stromal AnxA2 expression, are not significantly associated with DFS. HR: hazard ratio, CI: confidence interval.

### AnxA2 stromal expression is correlated with reduced overall survival

**Figure 4:**
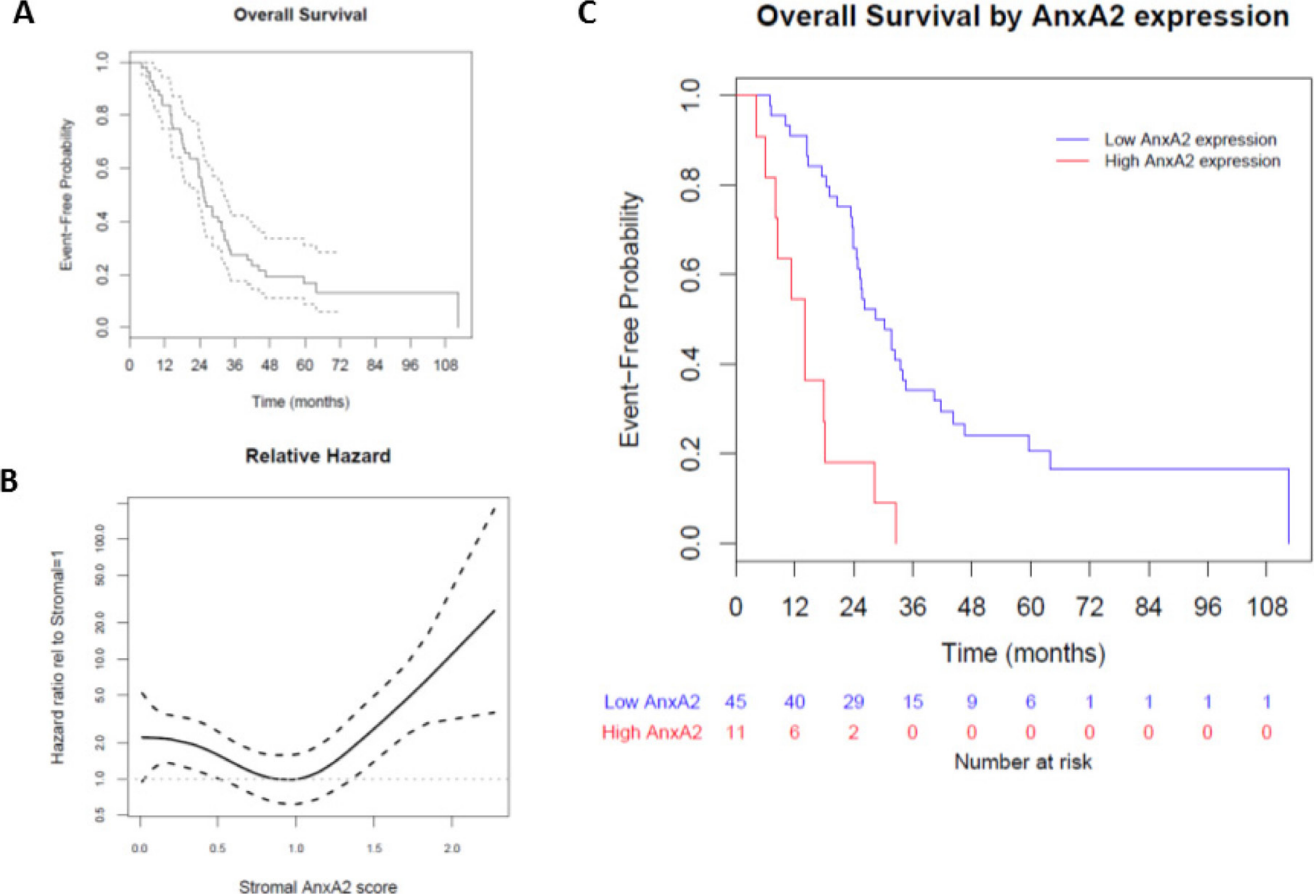
Effect of stromal AnxA2 score on overall survival We correlated the overall survival rates of the patients in our cohort with stromal AnxA2 expression. (**A**) Kaplan–Meier curve showing that the median OS in this cohort is 25.4 months (range, 23.7–32.4 months). (**B**) We used a Cox model with penalized smoothing splines of stromal AnxA2 score as covariates to evaluate log hazard at different covariate values. This plot shows the ratio of the hazard function for patients with different values of stromal AnxA2 score relative to that for patients with stromal AnxA2 score of 1.0. (**C**) Kaplan–Meier curves show that high stromal AnxA2 expression (>1.29) is associated with decreased DFS (*p* < 0.0001).

We evaluated the association between stromal AnxA2 expression scores with overall survival (OS) in our cohort. The median follow-up time for overall survival was 25.4 months (range 4.1–112.5 months). The Kaplan–Meier method estimates a median OS of 25.4 months (95% CI 23.7–32.4 months), and the 1-year and 2-year survival probabilities are estimated to be 0.84 (95% CI 0.75–0.94) and 0.57 (95% CI 0.45–0.71), respectively (Figure 4A). As before, we used Cox models with penalized smoothing splines to explore the effect of stromal AnxA2 expression on survival. The hazard ratio relative to stromal AnxA2 1 is shown in Figure 4B. Similar to Figure 3B for DFS, we observe a constant effect for stromal AnxA2 expression score less than 1 and an increasing trend in the risk of death for score greater than 1. When we use the same cutoff value of 1.29 (corresponding to the 80th percentile) for AnxA2 expression score, there is a significant difference in OS between high and low AnxA2 expression groups (14.0 vs. 30.2 months, *p* < 0.001, Figure 4C).

We then used multivariate Cox models to evaluate the adjusted effect of stromal AnxA2 expression on OS. Apart from the cutoff value of AnxA2 stromal expression, there was no significant association found between covariates and OS (Table 3).

**Table 3:** Multivariate analysis of factors related to overall survival

Variable	HR	95% CI	*P* value
AnxA2 score > 1.29	4.42	(1.84, 10.63)	0.001
Age > 60 years	0.94	(0.45, 1.96)	0.88
Female gender	1.89	(0.61. 5.89)	0.27
Tumor size > 3 cm	0.62	(0.18, 2.16)	0.46
Node positive	2.14	(0.69, 6.69)	0.19
Margin positive	0.96	(0.38, 2.44)	0.94
Grade ≥ 3	1.05	(0.52, 2.15)	0.88
Nodes ≥ 3	0.76	(0.33, 1.77)	0.52
Perineural invasion	0.53	(0.17, 1.70)	0.29
Vascular invasion	1.73	(0.78, 3.85)	0.18

Multivariate Cox modelling showing that clinical characteristics, excluding high stromal AnxA2 expression, do not affect OS. HR: hazard ratio, CI: confidence interval.

## DISCUSSION

We have shown that stromal expression of AnxA2 is associated with poor survival in patients with metastatic PDA. In addition, we have shown that AnxA2 plays an important role in the homing of PDA cells at distant metastatic sites. Specifically, when KPCA cells with restored AnxA2 function were administered in the hemi-spleen model, decreased survival was observed in mice expressing AnxA2 in the TME. This indicates that AnxA2 expression is necessary in both the TME and on tumor cells [12, 20].

In our cohort of patients who had undergone resection of pancreatic primary tumors, high stromal expression of AnxA2 was associated with a significantly reduced disease-free and overall survival. Consistent with our and other’s published studies delineating the role of AnxA2 in PDA neoplastic cells, Takano *et al* previously demonstrated that high expression of AnxA2 on the tumor cell surface (defined as >30% of tumor cells with positive staining) was correlated with rapid recurrence after adjuvant gemcitabine chemotherapy including decreased disease-free and overall survival in patients with resected PDA [22]. Here, we used automated analysis of stromal AnxA2 expression to distinguish high vs. low expression values and determined that high expression (defined as ≥80th percentile) was a factor that influenced survival outcomes in our cohort. This highlights the potential role of AnxA2 in cancer associated fibroblasts and the potential value for stromal AnxA2 to serve as a biomarker in patients with PDA.

The complex dynamic between components of the TME can be seen in the reverse Warburg effect where epithelial tumor cells are said to induce metabolic effects in neighboring stromal fibroblasts [28]. The notoriously dense fibrotic stroma in PDA results in a relatively hypovascular environment that poses a challenge to drug delivery. Several studies show concordance between tumoral and stromal expression of tumor markers suggesting that AnxA2 expression in both tumor cells and stromal cells might play a significant role in PDA metastasis [29, 30]. Previous studies have shown the role that AnxA2 plays in regulating the secretion of axon guidance molecules in PDA, but further studies are necessary to further delineate the specific roles of the stromal compartment in the AnxA2-PlxnD1-Sema3D pathway [20].

We have previously shown that AnxA2 expression is localized on the tumor cell surface and that cell surface expression increases as pancreatic lesions progress from precursor lesions (pancreatic intra-epithelial neoplasms) to invasive PDA [12]. This study demonstrated cytoplasmic staining of stromal fibroblasts in the PDA tumor microenvironment. AnxA2 is known to have key intracellular roles involving exocytosis, endocytosis and membrane trafficking [31–34]. Intracellular AnxA2 has also been shown to induce the expression of anti-apoptotic genes (including IL-6) leading to chemo-resistance in PDA cell lines [35]. It is plausible that the increased cytoplasmic expression of AnxA2 in fibroblasts enhances the secretory processes of axon guidance molecules, thereby assisting the processes of invasion and metastasis.

## CONCLUSIONS

In summary, this study shows that stromal AnxA2 expression may serve as a prognostic marker of survival in patients with PDA. It also provides further evidence of the importance of stromal components in the TME in mediating metastasis of PDA.

## MATERIALS AND METHODS

### Mouse models of PDA

Animals used in experiments were maintained according to the Animal Care and Use Committee guidelines of Johns Hopkins University.

The KPC mice, which is a genetically engineered mouse model of PDA through a knock-in of pancreatic-specific, conditional alleles of the *Kras*^G12D^ and *TP53*^R172H^ mutations on a mixed 129/SvJae/C57Bl/6 background [23], were backcrossed to the C57Bl/6 background as previously described [20]. AnxA2 homozygous knockout mice (*AnxA2*^−/−^) on a C57Bl/6 background [24] were crossed with the KPC mice to generate *Kras*^G12D^ *TP53*^R172H^ *Pdx-1-Cre*^+/+^ *AnxA2*^−/−^ mice (KPCA^−/−^ mice) [20].

The mouse hemi-spleen liver metastasis model has been previously described [25, 26]. In summary, spleens from anesthetized female C57Bl/6 mice ages 8 to 10 weeks were divided into two halves, and the halves were clipped. In total, 2 × 10^6^ PDA cells were injected into the splenic vessels (splenic artery and veins) through one hemi-spleen followed by a flush of PBS buffer. Following the injection, the splenic vessels draining the injected hemi-spleen were clipped, and the hemi-spleen was removed. The abdominal wall was sutured, and the skin was adapted using wound clips. All of the mice were followed twice daily for survival. Each experiment was repeated twice.

### KPC and KPCA^−/−^ primary epithelial tumor cell lines

The development of primary PDA cell lines from KPC or KPCA^−/−^ mice was described previously [20]. The cells were cultured in primary pancreas tumor medium (RPMI 1640, 10% FBS, 2 mM L-glutamine, 1% non-essential amino acids, 1 mM sodium pyruvate and 50 units/mL penicillin, 50 μg/mL streptomycin; Invitrogen) at 37°C, 5% CO_2_. AnxA2 expression was restored in the KPCA^−/−^ cell line as previously described [20].

### Human PDA specimens

All PDA specimens were obtained from consecutive patients undergoing pancreaticoduodenectomy and who received adjuvant chemoradiation therapy at Johns Hopkins Medical Institution (JHMI) in accordance with an Institutional Review Board (IRB)-approved protocol [27]. Only patients primarily followed at JHMI and whose archived paraffin-embedded tissue blocks were in good condition were included.

### Immunohistochemistry

Immunohistochemical staining for AnxA2 was performed on slides containing human pancreatic tumor tissue using a previously described protocol on an automated stainer (Leica Microsystems) [12].

### Image analysis and quantification

All IHC slides were digitally scanned using Aperio digital pathology whole slide scanner (Leich) and analyzed with ImageScope. Regions of stromal tissue were identified (average of 5 per slide) and the positive pixel count algorithm was used to quantify positive (brown) and negative (blue) pixels in the specified areas. The average sum of the positive pixels was normalized by the area of the delineated stromal regions to account for variations in the sizes of region measurements.

### Statistical analysis

Disease-free survival (DFS) was defined as the time from surgery to the first evidence of local or metastatic recurrent disease or death from any cause. Overall survival (OS) was defined as the time from surgery to death from any cause. Individuals were censored for DFS and OS at the date of the last known scan or contact, respectively. Kaplan–Meier curves were used to graphically compare the time-to-event outcomes based on stromal AnxA2 expression. Comparison between groups were made with log-rank tests and the effect of predictors were evaluated by using univariate and multivariate Cox proportional hazards regression models. Cox models with penalized polynomial smoothing splines were used to explore the nonlinear association between log-hazard and stromal AnxA2 expression. All analyses were performed using R Statistical Software (http://www.R-project.org; R Foundation for Statistical Computing, Vienna, Austria).

## SUPPLEMENTARY MATERIALS FIGURES



## References

[1] Siegel RL, Miller KD, Jemal A (2016). Cancer statistics, 2016. CA Cancer J Clin.

[2] Society AC Cancer Facts & Figures 2016.

[3] Chu GC, Kimmelman AC, Hezel AF, DePinho RA (2007). Stromal biology of pancreatic cancer. J Cell Biochem.

[4] Hwang RF, Moore T, Arumugam T, Ramachandran V, Amos KD, Rivera A, Ji B, Evans DB, Logsdon CD (2008). Cancer-associated stromal fibroblasts promote pancreatic tumor progression. Cancer Res.

[5] Rucki AA, Foley K, Zhang P, Xiao Q, Kleponis J, Wu A, Sharma R, Mo G, Liu A, Van Eyk J, Jaffee EM, Zheng L (2016). Heterogeneous stromal signaling within the tumor microenvironment controls the metastasis of pancreatic cancer. Cancer Res.

[6] Nyman DW, Campbell KJ, Hersh E, Long K, Richardson K, Trieu V, Desai N, Hawkins MJ, Von Hoff DD (2005). Phase I and pharmacokinetics trial of ABI-007, a novel nanoparticle formulation of paclitaxel in patients with advanced nonhematologic malignancies. J Clin Oncol.

[7] Olive KP, Jacobetz MA, Davidson CJ, Gopinathan A, McIntyre D, Honess D, Madhu B, Goldgraben MA, Caldwell ME, Allard D, Frese KK, Denicola G, Feig C (2009). Inhibition of Hedgehog signaling enhances delivery of chemotherapy in a mouse model of pancreatic cancer. Science.

[8] Ozdemir BC, Pentcheva-Hoang T, Carstens JL, Zheng X, Wu CC, Simpson TR, Laklai H, Sugimoto H, Kahlert C, Novitskiy SV, De Jesus-Acosta A, Sharma P, Heidari P (2014). Depletion of carcinoma-associated fibroblasts and fibrosis induces immunosuppression and accelerates pancreas cancer with reduced survival. Cancer Cell.

[9] Rhim AD, Oberstein PE, Thomas DH, Mirek ET, Palermo CF, Sastra SA, Dekleva EN, Saunders T, Becerra CP, Tattersall IW, Westphalen CB, Kitajewski J, Fernandez-Barrena MG (2014). Stromal elements act to restrain, rather than support, pancreatic ductal adenocarcinoma. Cancer Cell.

[10] Whatcott CJ, Han H, Posner RG, Hostetter G, Von Hoff DD (2011). Targeting the tumor microenvironment in cancer: why hyaluronidase deserves a second look. Cancer Discov.

[11] Hingorani SR, Harris WP, Beck JT, Berdov BA, Wagner SA, Pshevlotsky EM, Tjulandin SA, Gladkov OA, Holcombe RF, Korn R, Raghunand N, Dychter S, Jiang P (2016). Phase Ib Study of PEGylated Recombinant Human Hyaluronidase and Gemcitabine in Patients with Advanced Pancreatic Cancer. Clin Cancer Res.

[12] Zheng L, Foley K, Huang L, Leubner A, Mo G, Olino K, Edil BH, Mizuma M, Sharma R, Le DT, Anders RA, Illei PB, Van Eyk JE (2011). Tyrosine 23 phosphorylation-dependent cell-surface localization of annexin A2 is required for invasion and metastases of pancreatic cancer. PLoS One.

[13] Lutz E, Yeo CJ, Lillemoe KD, Biedrzycki B, Kobrin B, Herman J, Sugar E, Piantadosi S, Cameron JL, Solt S, Onners B, Tartakovsky I, Choi M (2011). A lethally irradiated allogeneic granulocyte-macrophage colony stimulating factor-secreting tumor vaccine for pancreatic adenocarcinoma. A Phase II trial of safety, efficacy, and immune activation. Ann Surg.

[14] Farnaes L, Ditzel HJ (2003). Dissecting the cellular functions of annexin XI using recombinant human annexin XI-specific autoantibodies cloned by phage display. J Biol Chem.

[15] Cui HY, Wang SJ, Miao JY, Fu ZG, Feng F, Wu J, Yang XM, Chen ZN, Jiang JL (2016). CD147 regulates cancer migration via direct interaction with Annexin A2 and DOCK3-beta-catenin-WAVE2 signaling. Oncotarget.

[16] Wang CY, Chen CL, Tseng YL, Fang YT, Lin YS, Su WC, Chen CC, Chang KC, Wang YC, Lin CF (2012). Annexin A2 silencing induces G2 arrest of non-small cell lung cancer cells through p53-dependent and -independent mechanisms. J Biol Chem.

[17] Yang T, Peng H, Wang J, Yang J, Nice EC, Xie K, Huang C (2013). Prognostic and diagnostic significance of annexin A2 in colorectal cancer. Colorectal Dis.

[18] Hajjar KA, Krishnan S (1999). Annexin II: a mediator of the plasmin/plasminogen activator system. Trends Cardiovasc Med.

[19] Cavallo-Medved D, Rudy D, Blum G, Bogyo M, Caglic D, Sloane BF (2009). Live-cell imaging demonstrates extracellular matrix degradation in association with active cathepsin B in caveolae of endothelial cells during tube formation. Exp Cell Res.

[20] Foley K, Rucki AA, Xiao Q, Zhou D, Leubner A, Mo G, Kleponis J, Wu AA, Sharma R, Jiang Q, Anders RA, Iacobuzio-Donahue CA, Hajjar KA (2015). Semaphorin 3D autocrine signaling mediates the metastatic role of annexin A2 in pancreatic cancer. Sci Signal.

[21] Vishwanatha JK, Chiang Y, Kumble KD, Hollingsworth MA, Pour PM (1993). Enhanced expression of annexin II in human pancreatic carcinoma cells and primary pancreatic cancers. Carcinogenesis.

[22] Takano S, Togawa A, Yoshitomi H, Shida T, Kimura F, Shimizu H, Yoshidome H, Ohtsuka M, Kato A, Tomonaga T, Nomura F, Miyazaki M (2008). Annexin II overexpression predicts rapid recurrence after surgery in pancreatic cancer patients undergoing gemcitabine-adjuvant chemotherapy. Ann Surg Oncol.

[23] Hingorani SR, Wang L, Multani AS, Combs C, Deramaudt TB, Hruban RH, Rustgi AK, Chang S, Tuveson DA (2005). Trp53R172H and KrasG12D cooperate to promote chromosomal instability and widely metastatic pancreatic ductal adenocarcinoma in mice. Cancer Cell.

[24] Ling Q, Jacovina AT, Deora A, Febbraio M, Simantov R, Silverstein RL, Hempstead B, Mark WH, Hajjar KA (2004). Annexin II regulates fibrin homeostasis and neoangiogenesis *in vivo*. J Clin Invest.

[25] Jain A, Slansky JE, Matey LC, Allen HE, Pardoll DM, Schulick RD (2003). Synergistic effect of a granulocyte-macrophage colony-stimulating factor-transduced tumor vaccine and systemic interleukin-2 in the treatment of murine colorectal cancer hepatic metastases. Ann Surg Oncol.

[26] Soares KC, Foley K, Olino K, Leubner A, Mayo SC, Jain A, Jaffee E, Schulick RD, Yoshimura K, Edil B, Zheng L (2014). A preclinical murine model of hepatic metastases. J Vis Exp.

[27] Bever KM, Sugar EA, Bigelow E, Sharma R, Laheru D, Wolfgang CL, Jaffee EM, Anders RA, De Jesus-Acosta A, Zheng L (2015). The prognostic value of stroma in pancreatic cancer in patients receiving adjuvant therapy. HPB (Oxford).

[28] Pavlides S, Whitaker-Menezes D, Castello-Cros R, Flomenberg N, Witkiewicz AK, Frank PG, Casimiro MC, Wang C, Fortina P, Addya S, Pestell RG, Martinez-Outschoorn UE, Sotgia F (2009). The reverse Warburg effect: aerobic glycolysis in cancer associated fibroblasts and the tumor stroma. Cell Cycle.

[29] Huang J, Fan X, Wang X, Lu Y, Zhu H, Wang W, Zhang S, Wang Z (2015). High ROR2 expression in tumor cells and stroma is correlated with poor prognosis in pancreatic ductal adenocarcinoma. Sci Rep.

[30] Kasashima H, Yashiro M, Kinoshita H, Fukuoka T, Morisaki T, Masuda G, Sakurai K, Kubo N, Ohira M, Hirakawa K (2014). Lysyl oxidase-like 2 (LOXL2) from stromal fibroblasts stimulates the progression of gastric cancer. Cancer Lett.

[31] Sarafian T, Pradel LA, Henry JP, Aunis D, Bader MF (1991). The participation of annexin II (calpactin I) in calcium-evoked exocytosis requires protein kinase C. J Cell Biol.

[32] Emans N, Gorvel JP, Walter C, Gerke V, Kellner R, Griffiths G, Gruenberg J (1993). Annexin II is a major component of fusogenic endosomal vesicles. J Cell Biol.

[33] Morel E, Gruenberg J (2007). The p11/S100A10 light chain of annexin A2 is dispensable for annexin A2 association to endosomes and functions in endosomal transport. PLoS One.

[34] Babiychuk EB, Draeger A (2000). Annexins in cell membrane dynamics. Ca(2+)-regulated association of lipid microdomains. J Cell Biol.

[35] Jung H, Kim JS, Kim WK, Oh KJ, Kim JM, Lee HJ, Han BS, Kim DS, Seo YS, Lee SC, Park SG, Bae KH (2015). Intracellular annexin A2 regulates NF-kappaB signaling by binding to the p50 subunit: implications for gemcitabine resistance in pancreatic cancer. Cell Death Dis.

