# Supplementary material for: Stromal Annexin A2 expression is predictive of decreased survival in pancreatic cancer
Source: Oncotarget. 2017 Nov 15;8(63):106405–14. doi: 10.18632/oncotarget.22433 (PMC5739743; doi:10.18632/oncotarget.22433)
Supplement: Supplementary file 1 [file oncotarget-08-106405-s001.pdf]

Stromal Annexin A2 expression is predictive of decreased survival in pancreatic cancer

SUPPLEMENTARY MATERIALS

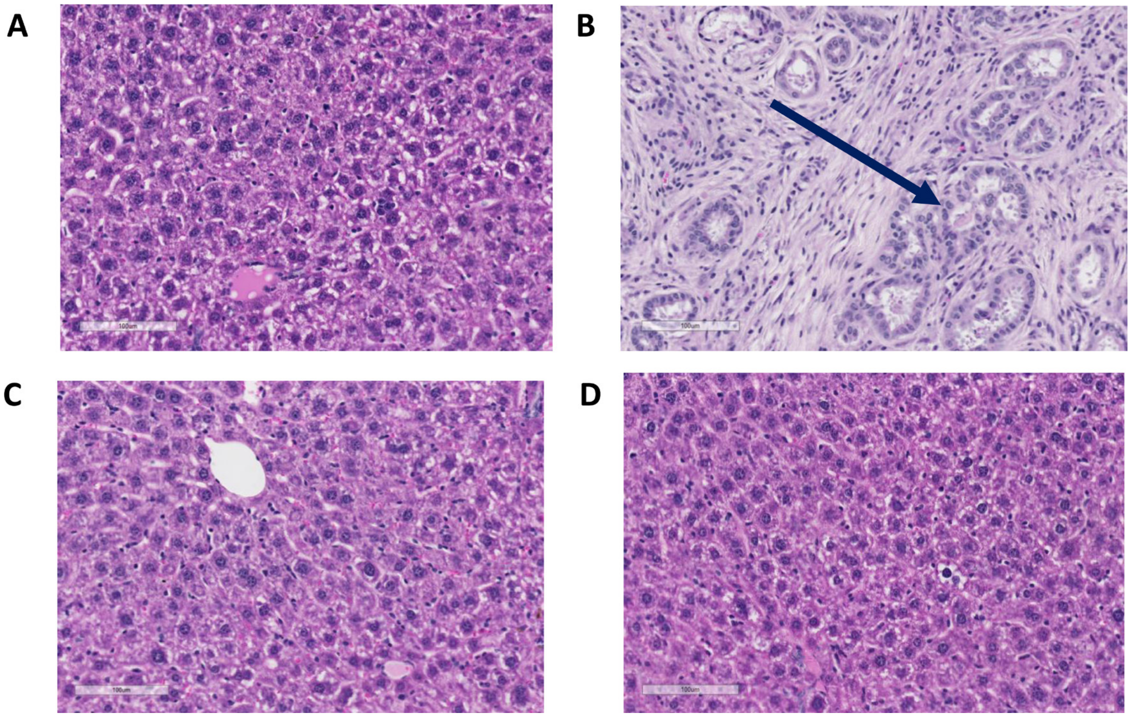

Supplementary Figure 1: Histological appearance of livers in the hemi-splenectomy model. Representative H&E images of the liver from animals injected with pancreatic tumor cells in the hemi-splenectomy model. (**A**) B6 mouse injected with KPCA GFP$^+$ cells showing absence of tumor cells. (**B**) B6 mouse with KPCA + AnxA2 cells denoting presence of tumor (black arrows indicate tumor). (**C**) AnxA2 KO mouse injected with KPCA GFP$^+$ cells showing absence of tumor cells. (**D**) AnxA2 KO mouse injected with KPCA + AnxA2 cells showing absence of tumor cells.

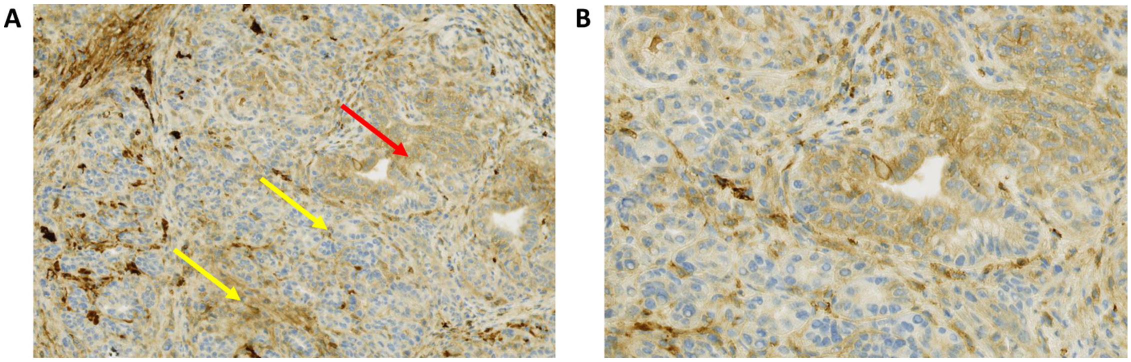

Supplementary Figure 2: Stromal expression of AnxA2 in murine KPC cells. Representative images of AnxA2 staining from animals injected with pancreatic tumor cells in the hemi-splenectomy model. (**A**) KPC tumor (red arrow) with stromal fibroblast staining (yellow arrows). (**B**) shows same image at higher magnification (20×).
